# Ageing combines CD4 T cell lymphopenia in secondary lymphoid organs and T cell accumulation in gut associated lymphoid tissue

**DOI:** 10.1186/1742-4933-11-8

**Published:** 2014-05-08

**Authors:** Kim Zita Martinet, Stéphane Bloquet, Christine Bourgeois

**Affiliations:** 1 INSERM U1012, Faculté de Médecine Paris-Sud, 63 rue Gabriel Péri, 94276 Le Kremlin-Bicêtre, France; 2 Univ Paris-Sud, UMR-S1012, Le Kremlin-Bicêtre, France; 3 Animalerie centrale, Faculté de Médecine Paris-Sud, Univ Paris-Sud, Le Kremlin-Bicêtre, France

**Keywords:** Immunology, T cell, T cell lymphopenia, T cell homeostasis, Immunosenescence, Ageing, Aging, Age, GALT, MALT, CD4 T cell lymphopenia, CD4 T cell homeostasis

## Abstract

**Background:**

CD4 T cell lymphopenia is an important T cell defect associated to ageing. Higher susceptibility to infections, cancer, or autoimmune pathologies described in aged individuals is thought to partly rely on T cell lymphopenia. We hypothesize that such diverse effects may reflect anatomical heterogeneity of age related T cell lymphopenia. Indeed, no data are currently available on the impact of ageing on T cell pool recovered from gut associated lymphoid tissue (GALT), a crucial site of CD4 T cell accumulation.

**Results:**

Primary, secondary and tertiary lymphoid organs of C57BL/6 animals were analysed at three intervals of ages: 2 to 6 months (young), 10 to 14 months (middle-aged) and 22 to 26 months (old). We confirmed that ageing preferentially impacted CD4 T cell compartment in secondary lymphoid organs. Importantly, a different picture emerged from gut associated mucosal sites: during ageing, CD4 T cell accumulation was progressively developing in colon and small intestine lamina propria and Peyer’s patches. Similar trend was also observed in middle-aged SJL/B6 F1 mice. Interestingly, an inverse correlation was detected between CD4 T cell numbers in secondary lymphoid organs and colonic lamina propria of C57BL/6 mice whereas no increase in proliferation rate of GALT CD4 T cells was detected. In contrast to GALT, no CD4 T cell accumulation was detected in lungs and liver in middle-aged animals. Finally, the concomitant accumulation of CD4 T cell in GALT and depletion in secondary lymphoid organs during ageing was detected both in male and female animals.

**Conclusions:**

Our data thus demonstrate that T cell lymphopenia in secondary lymphoid organs currently associated to ageing is not sustained in gut or lung mucosa associated lymphoid tissues or non-lymphoid sites such as the liver. The inverse correlation between CD4 T cell numbers in secondary lymphoid organs and colonic lamina propria and the absence of overt proliferation in GALT suggest that marked CD4 T cell decay in secondary lymphoid organs during ageing reflect redistribution of CD4 T cells rather than generalized CD4 T cell decay. Such anatomical heterogeneity may provide an important rationale for the diversity of immune defects observed during ageing.

## Background

Increased susceptibility to infections, cancer [1,2] or some auto-immune pathologies [3,4] are immune defects commonly associated to ageing [5]. The impact of ageing on T cell compartment is commonly defined by reduction of thymic production, T cell lymphopenia, increased of effector/memory T cells subsets [6,7]. Additionally, intrinsic defects affecting T cell responsiveness have been described [8]: altered cell membrane fluidity [9], alteration of cell surface expression of co-stimulatory molecules and cytokine receptors, molecular and transcriptional changes [10]. T cell lymphopenia, i.e. decrease in T cell number, has been extensively described during human [11-13] and mice ageing [14,15] and is considered as an important cofactor of age related immune defects. However, the mechanisms by which T cell lymphopenia contribute both to defective and/or exacerbated immune responses in context of infection or autoimmunity respectively remain uncertain. Two opposing immune alterations have been essentially associated to T cell lymphopenia so far. A first mechanism is the loss of clonal diversity [16-20] presumably leading to the inability of elderly to respond to certain pathogens. It is commonly related to progressive decrease in thymic export [21-23] although such mechanism is probably not exclusive [24-27]. Importantly, loss of diversity is not consistently described in human analyses and remains to be fully characterized [28]. The second alteration commonly related to age is the higher proportion of effector/memory cells among conventional T cells in aged individuals [29]. The age related skewing of naïve T cells towards effector and/or memory T cells is thought to reflect both loss of naïve T cell production and lymphopenia induced proliferation (LIP) of naïve T cells [30,31]. It has been claimed that such exacerbated immune responses may lead to auto-immune pathologies [3,4]. However, LIP essentially occurred in context of severe lymphopenia (such as constitutively lymphopenic hosts) and may not develop in context of partial lymphopenia such as observed in ageing [14,32]. Thus, the exact relationship between diversity of age-related immune defects and T cell lymphopenia remains under debate. We hypothesize that age-related T cell lymphopenia develops differently in secondary lymphoid organs compared to tertiary lymphoid organs and non-lymphoid tissues. Indeed, T cell lymphopenia has been essentially described in secondary lymphoid organs and blood. However, very little information is currently available on T cell compartment in mucosa associated lymphoid tissues (MALT) such as gut or lung associated lymphoid tissue or non-lymphoid tissues. We focused on gut associated lymphoid tissue (GALT) that is presumably highly relevant to CD4 T cell homeostasis [33] and readdressed T cell lymphopenia during ageing by performing an exhaustive analysis of the impact of ageing on T cell compartment integrating anatomical location. We analysed CD4 and CD8 T cell compartments in secondary (i.e. mesenteric lymph nodes, superficial lymph nodes, and spleen either globally or separately), tertiary lymphoid organs (as MALT) and non-lymphoid tissue (liver) for different ages ranges (young (2–6 months old), middle-aged (10–14 months old) and old C57BL/6 animals (22–26 months old)).

We confirmed that ageing preferentially affected CD4 T cell compartment in secondary lymphoid organs. We demonstrated that ageing induces concomitantly CD4 T cell decay in secondary lymphoid organs (and notably superficial lymph nodes) and increase of CD4 T cell numbers in the lamina propria of colon and small intestine and Peyer’s patches. Such aged related CD4 T cell accumulation was locally restricted since liver or lungs did not show any difference with age in CD4 T cell absolute numbers. Interestingly, an inverse association was detected between CD4 T cell numbers recovered in secondary lymphoid organs and in colonic lamina propria. Secondly, we demonstrated that T cell accumulation in GALT was not related to higher proliferation of GALT CD4 T cells during ageing. Collectively these data suggest that CD4 T cell lymphopenia commonly described in secondary lymphoid organs (and blood) during ageing may reflect altered redistribution of CD4 T cells rather than extensive depletion. Such anatomical heterogeneity may provide an important rationale for the diversity of immune defects observed during ageing and lead to drastically re-evaluate the notion of age related T cell lymphopenia.

## Results

### Differential CD4 and CD8 decay in lymphoid organs depending on age

**Figure 1:**
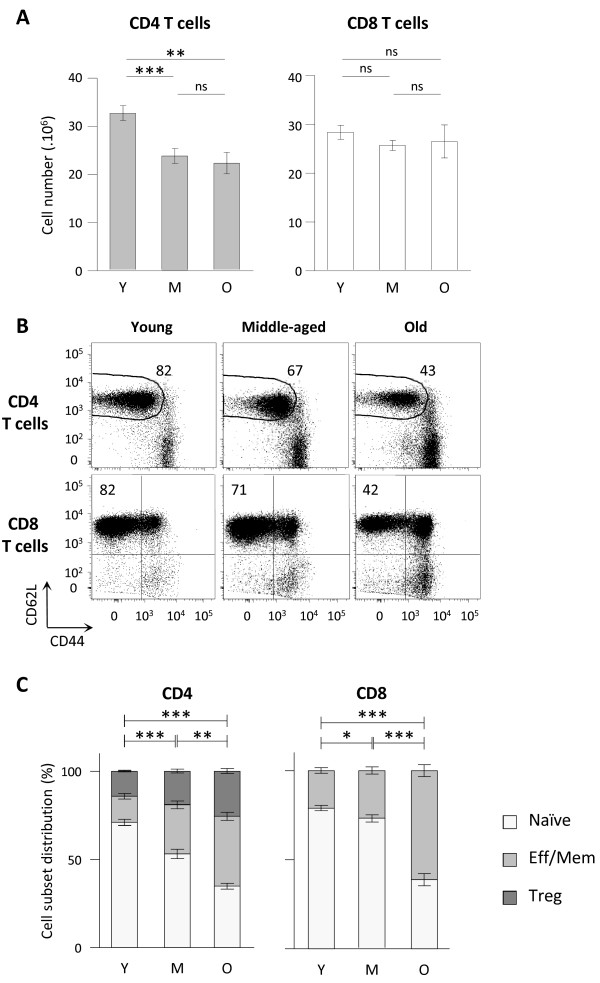
**CD4 but not CD8 T cell decay in secondary lymphoid organs during ageing. (A)** Total CD4 and CD8 T cell numbers recovered from pooled spleen and lymph nodes. Numeration and FACS analyses of secondary lymphoid organs were performed on young (2–6 months (Y); n = 30), middle-aged (10–14 months (M); n = 20) and old (22–26 months (O); n = 12) C57BL/6 mice. **(B)** Representative dot plots of CD62L and CD44 expression on FOXP3- CD4 T cells (conventional CD4 T cells) and CD8 T cells recovered from spleen of young, middle-aged and old C57BL/6 mice. **(C)** Naïve, effector/memory or regulatory of CD4 and CD8 T cell distributions in pooled spleen and lymph nodes from young (n = 20), middle-aged (n = 10) and old (n = 10) C57BL/6 mice. Naïve CD4 T cells were identified as CD45+ TCRβ+ CD4+ FOXP3- CD62L+ CD44low cells, naïve CD8 T cells as CD45+ TCRβ+ CD8α+ CD62L+ CD44- cells, effector/memory CD4 T cells as CD45+ TCRβ+ CD4+ FOXP3- CD62L- CD44high, effector/memory CD8 T cells as CD45+ TCRβ+ CD8α+ non CD62L+CD44- and regulatory CD4 T cells (Treg) as CD45+ TCRβ+ CD4+ FOXP3+. Cumulative results show the mean ± SEM of absolute numbers. P values indicate statistical difference between effector/memory proportion depending on age as followed: ns, non-significant; *, p < 0.05; **, p < 0.01; ***, p < 0.001 (Student’s *t* test).

**Figure 2:**
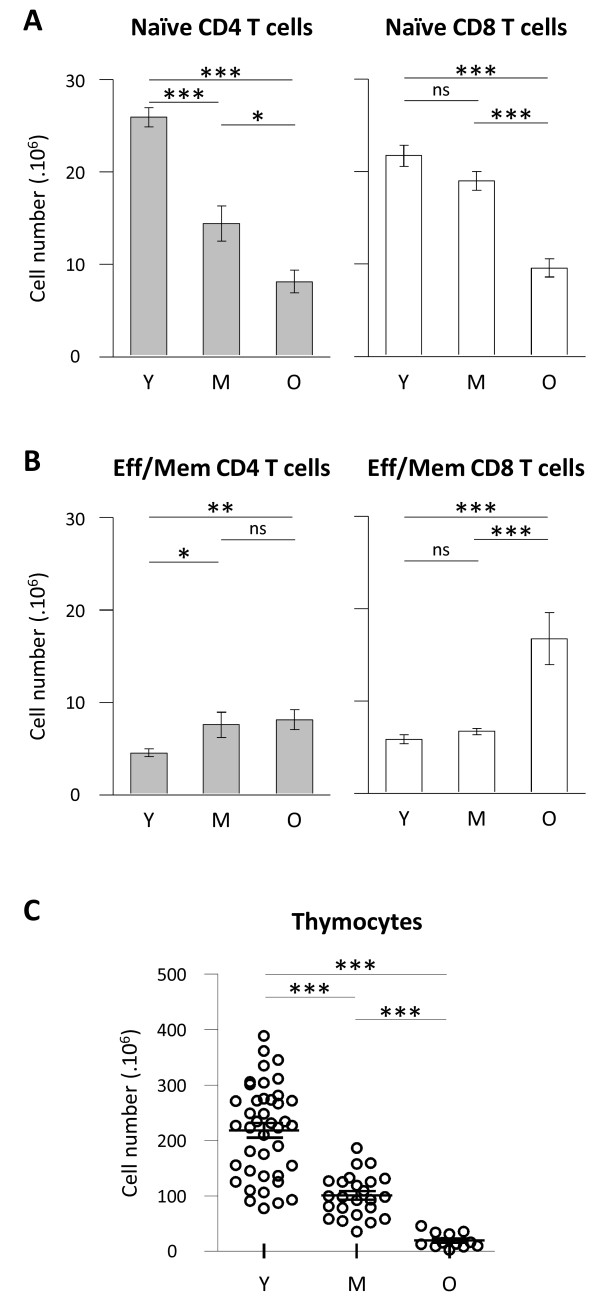
**Naïve and effector/memory CD4 and CD8 absolute numbers in secondary lymphoid organs during ageing.** Numeration and FACS analyses were performed on spleen and lymph nodes from young, middle-aged and old C57BL/6 mice as described in Figure 1. **(A, B)** Absolute numbers of naïve **(A)** and effector/memory **(B)** CD4 and CD8 T cells recovered in secondary lymphoid organs. **(C)** Thymocyte numbers. Numerations were performed on young (n = 10 to 30), middle-aged (n = 10 to 20) and old (n = 10 to 12) C57BL/6 mice. For each experiment, comparison of young animals to middle-aged and/or old animals was simultaneously performed. Cumulative results show the mean ± SEM of absolute numbers. Statistical significance (Student’s *t* test) is shown: ns, non-significant; *, p < 0.05; **, p < 0.01; ***, p < 0.001.

C57BL/6 animals were analysed at three intervals of ages: 2 to 6 months (young), 10 to 14 months (middle-aged) and 22 to 26 months (old). We first determined the absolute number of CD45+ TCRβ+ CD4+ (CD4 T cells) or CD45+ TCRβ+ CD8α+ (CD8 T cells) lymphocytes recovered from secondary lymphoid organs (spleen and lymph nodes) at different ages (Figure 1A). We observed a significant decrease in the number of CD4 T cells in lymphoid organs in “middle-aged” and “old” mice compared to “young” animals. No significant decay was further detected between 10–14 months and 22–26 months old mice. In contrast, we did not detect variations in CD8 T cell numbers whatever the age interval considered. We thus confirmed a differential behaviour of CD4 and CD8 T cells depending on the age [15,34-36]. Ageing is commonly associated to a shift of naïve and effector/memory T cell subsets proportion towards effector/memory T cells [25,29,37,38]. We thus evaluated whether the proportion of naïve and effector/memory cell pools were equally affected in CD4 and CD8 T cell subsets during ageing. Analysing L-selectin (CD62L), a lymphoid homing molecule, and CD44 expression, we identified naïve and effector memory T cell subsets in secondary lymphoid organs, as exemplified on splenic T cells in Figure 1B. Analyses on CD4 T cells were performed on FOXP3- cells to exclude regulatory T cells (FOXP3+) that may exhibit different homeostasis compared to the so-called conventional FOXP3- CD4 T cells. Increasing frequencies of both effector/memory CD4 and CD8 T cells were already detectable when comparing middle-aged to young animals (Figure 1C). However the amplitude of skewing differed when considering CD4 and CD8 T cell subsets: effector/memory CD4 T shift was progressively developing from young to old animals, whereas effector/memory CD8 T shift was mildly increased in middle-aged animals, and became more intense from middle-aged to old animals. Thus, whereas CD4 and CD8 T cell numbers are differently affected during ageing, increasing proportion of effector/memory T cells are detected in both CD4 and CD8 T cells. We further investigated this apparent discrepancy by analysing naïve and effector/memory T cell numbers. Naïve CD4 T cell numbers were significantly different between young and middle-aged mice whereas naïve CD8 T cell numbers remain statistically stable in the same interval (Figure 2A). However, both naive CD4 and CD8 T cell numbers were significantly decreased in old mice compared to young animals. We thus observed a different impact of ageing on CD4 and CD8 T cells among naïve T cell compartment: naïve CD4 T cell compartment being affected more rapidly than naïve CD8 T cell pool. Reduction in naïve CD4 T cell numbers in lymphoid organs essentially corroborated the decay observed in total CD4 T cells. However, the drastic decay observed in naïve CD8 T cells in old mice was not reflected in total CD8 T cell numbers. We next considered the evolution of effector/memory T cell numbers: the number of effector/memory CD4 T cells (TCRβ+ CD4+ FOXP3- CD62L- CD44high cells) significantly increased in middle-aged and old mice, albeit with low amplitude, compared to young animals (Figure 2B). Evolution of regulatory FOXP3+ CD4+ T cells (Treg) numbers exhibited a similar profile than effector/memory CD4 T cells numbers (not shown). Regarding effector/memory CD8 T cell numbers (TCRβ+ CD8α+ non CD62L+CD44- cells) recovered from lymphoid organs, no significant difference was detected between young and middle-aged animals. However, a significant increase was detected when comparing old animals to young or middle-aged groups (Figure 2B). As a control for ageing, we finally determined thymocyte numbers in the three groups of age. As expected, reduction in thymocyte numbers, a clear hallmark of T cell ageing, was significantly detected in middle-aged mice and further aggravated in old mice (Figure 2C).

Collectively, analysing naïve and effector/memory absolute numbers provided interesting insights on the shift of naïve T cells towards effector/memory T cells during ageing. We observed that physiological ageing is not equally affecting CD4 and CD8 T cell pools. Total CD4 T cell decay reflected massive reduction of naïve CD4 T cells occurring in middle-aged animals combined to a mild increase of effector/memory CD4 T cells in old animals. A different timeline emerged when considering CD8 T cell compartment: naïve and effector/memory CD8 T cells numbers were essentially not affected in middle-aged animals in contrast to older animals who exhibited clear naïve CD8 T cell decay and increase in effector/memory CD8 T cells.

### T cell decay differed depending on the second lymphoid organs considered

**Figure 3:**
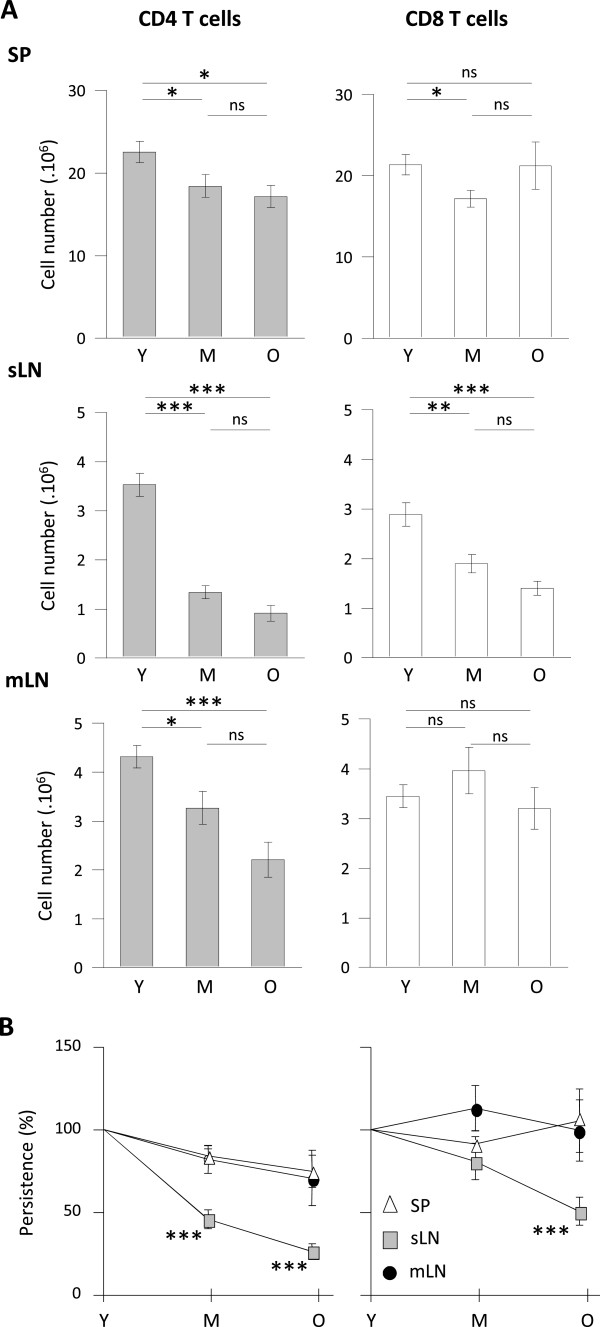
**Age related T cell lymphopenia differently affects secondary lymphoid organs. (A)** CD4 and CD8 T cell numbers recovered from spleen (SP), superficial (inguinal, brachial and axillary lymph nodes (sLN)) and mesenteric (mLN) lymph nodes. Numeration and FACS analyses of each secondary lymphoid organs were performed from young (Y) (n = 30), middle-aged (M) (n = 20) and old (O) (n = 12) C57BL/6 mice. **(B)** CD4 and CD8 T cell persistence in each secondary lymphoid organ. Age related persistence was expressed as the percentage of residual T cells recovered in either middle-aged or old mice compared to values recovered from young animals [older absolute number / young absolute number x 100]. Graphs show mean ± SEM of absolute numbers of independent experiments comparing young animals to middle-aged and/or old animals. Statistical difference between sLN and SP T cell recovery depending of age is shown (Student’s *t* test): ns, non-significant; *, p < 0.05; **, p < 0.01; ***, p < 0.001.

Because some contradictions emerged from data on T cell numbers recovered from lymph nodes and/or spleen [14,39], we next ascertain whether differential behaviour of CD4 and CD8 T cells was homogenous in all secondary lymphoid organs. When considering separately spleen, mesenteric lymph nodes and superficial lymph nodes (i.e. axillary, brachial and inguinal lymph nodes), CD4 T cell decay was detected in all organs when comparing middle-aged or old mice to young animals (Figure 3A left). However, the amplitude differed: CD4 T cells from superficial lymph nodes appeared more affected than those in mesenteric lymph nodes and spleen. Because total CD8 T cell numbers were essentially preserved in pooled secondary lymphoid organs analysis, we were not expecting a major difference in secondary lymphoid organs considered individually. As expected, numbers of CD8 T cells recovered in the spleen and mesenteric lymph node were essentially not affected, as mice grew older. However, superficial lymph nodes exhibited a different profile revealing a significant decay in the numbers of CD8 (Figure 3A right). In conclusion, T cell distribution was gradually affected depending on the lymphoid organs considered: splenic cells appeared mildly affected; mesenteric lymph nodes exhibited partial T cell lymphopenia; T cell lymphopenia was more marked in superficial lymph nodes. To directly compare T cell decay in each secondary lymphoid structure considered, we presented the percentage of residual CD4 and CD8 T cells in middle-aged and old animals compared to young mice (Figure 3B). The percentage of residual CD4 T cells at 10–14 months (middle-age) was significantly lower in the superficial lymph nodes compared to mesenteric lymph nodes and spleen (p < 0.01 and <0.001 respectively). CD4 T cell decay in mesenteric lymph nodes resembled those observed in spleen. Stronger decay in superficial lymph nodes compared to mesenteric lymph nodes or spleen was also found in 22–26 months old mice (p < 0.05 and <0.001 respectively). Similarly, the specific impact of ageing in superficial lymph nodes was detected among CD8 T cells, although detectable solely in old animals (p < 0.05 and p < 0.001 when comparing to mLN and SP respectively). We thus observed that age related T cell lymphopenia is exacerbated in superficial lymph nodes, whereas T cells in mesenteric lymph nodes and spleen are better preserved.

### Age associated T cell accumulation in gut associated lymphoid tissue during ageing

**Figure 4:**
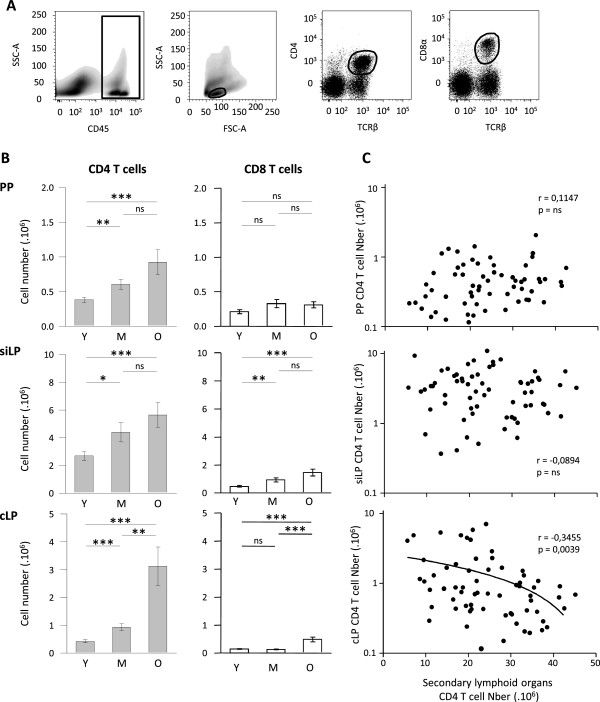
**CD4 and CD8 T cell absolute numbers increased during ageing in gut associated lymphoid tissues. (A)** Gating strategy to identify CD4 and CD8 T cells. Dot plots shows cells isolated from small intestine lamina propria of a 23 months old C57BL/6 mouse. CD45 expressing cells were first selected, then lymphocytes gate was determined to finally discriminate TCRβ+ CD4+ cells and TCRβ+ CD8α+ cells. **(B)** Graphs show total CD4 (**left**) and CD8 (**right**) T cell numbers recovered from Peyer’s patches (PP), small intestine lamina propria (siLP) and colonic lamina propria (cLP) during ageing. Numeration and FACS analysis were performed on PP, siLP and cLP from young (Y) (n = 20), middle-aged (M) (n = 15) and old (O) (n = 10) C57BL/6 mice. Cumulative results show the mean ± SEM of absolute numbers recovered from independent experiment comparing young, middle-aged and/or old animals. Statistical significance (Student’s *t* test) is shown: ns, non-significant; *, p < 0.05; **, p < 0.01; ***, p < 0.001. **(C)** Association between mucosal CD4 T cell numbers (PP, siLP and cLP respectively) and secondary lymphoid organs (pooled mesenteric, axillary, brachial and inguinal lymph nodes and spleen) CD4 T cell numbers was evaluated using Spearman test. Spearman r and p value are indicated in each graph.

The obvious dichotomy between superficial and mesenteric lymph nodes led us to further investigate gut associated lymphoid structure. Because CD4 T cells in the gut have been essentially located in the lamina propria, we isolated T lymphocytes from colonic lamina propria (cLP), small intestine lamina propria (siLP) and associated lymphoid structures: Peyer’s patches. The gating strategy is shown in Figure 4A. We studied the dynamics of T cell numbers in these structures depending on the age. In Peyer’s patches, CD4 T cell numbers increased with age but absolute CD8 T cell numbers remained constant through ageing. In siLP, both CD4 and CD8 absolute numbers progressively increased from young to middle-aged or old mice (Figure 4B). In cLP, we observed a different dynamic: increase in CD4 and CD8 T cell numbers was predominantly observed between 10–14 months and 22–26 months (Figure 4B). We performed a similar set of experiment in a different strain background using B6/SJL F1 animals. Comparing “young” and “intermediate” B6/SJL F1 animals, we detected decreased thymocytes (157.9 +/−13.5 10^6^ versus 15.1 +/−5.6 10^6^ thymocytes in young and middle-aged animals respectively; <0.0001). Regarding gut associated lymphoid tissue (GALT), an increase in CD4 T cell numbers in Peyer’s patches was detected (0.35 +/−0.09 10^6^ versus 0.85 +/−0.15 10^6^ CD4 T cells in young and middle-aged animals respectively; p 0.0128) and preserved CD4 T cell counts were observed in cLP and siLP. Thus we confirmed in a different strain model that T cell lymphopenia developing in lymphoid organs was not recapitulated in GALT and even associated to CD4 T cell accumulation at some sites.

### Association between CD4 T cell lymphopenia in secondary lymphoid organs and accumulation in gut associated lymphoid tissue

Simultaneous accumulation of CD4 T cell at mucosal sites and the development of T cell lymphopenia at lymphoid sites raised the question of a direct association between these two phenomena during ageing. Indeed, a strong inverse association was observed between CD4 T cell numbers in colonic lamina propria (cLP) sites and lymphoid sites (Figure 4C). To note, the association was not detected when considering small intestine lamina propria (siLP) or Peyer’s patches (PP) presumably due to the limited variation in numbers observed in these organs. Indeed, ageing induced a 6-fold increase in CD4 T cells in cLP but a 2-fold increase in siLP and PP.

**Figure 5:**
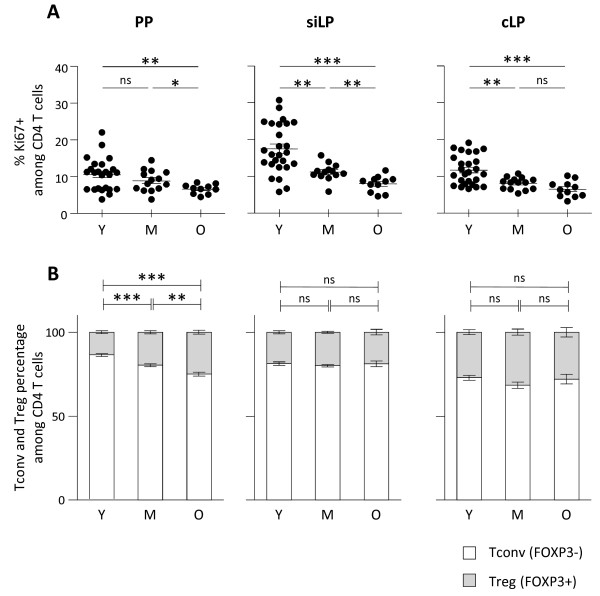
**No local proliferation, nor preferential Treg induction in ageing GALT. (A)** Proliferative status of CD4 T cells in the GALT. Percentage of Ki67 expressing cells were determined on CD4 T cells recovered from PP, siLP and cLP from young (Y) (n = 24), middle-aged (M) (n = 12) and old (O) (n = 10) C57BL/6 mice. **(B)** Proportion of FOXP3 non-expressing (Tconv) and FOXP3 expressing (Treg) CD4 T cells in GALT from young (n = 25), middle-aged (n = 15) and old (n = 10) C57BL/6 mice. Cumulative results show the mean ± SEM of absolute numbers recovered from independent experiments comparing young, middle-aged and aged animals. Statistical significance (Student’s *t* test) is shown: ns, non-significant; *, p < 0.05; **, p < 0.01.

To evaluate whether such accumulation in GALT is reflecting local proliferation or, instead, recruitment to the site, we determined Ki67 expression in CD4 T cells recovered from GALT. We could not detect any increase in Ki67 expression in any of the three gut associated lymphoid structures during ageing. A significant reduction of Ki67 positive CD4 T cells was even detected in all three organs at old ages. Reduced proliferation was already detectable at middle age in siLP and cLP, suggesting local proliferation was not responsible for such accumulation (Figure 5A). As a control, Ki67 expression was also assessed in secondary lymphoid organs, which exhibited significant increase in proliferative fraction (data not shown). Because GALT has been described as a favourable environment for regulatory T cells (Treg) induction, we finally ascertain that CD4 T cell accumulation was not directly reflecting specific accumulation or production of FOXP3 expressing Treg. No or mild increase in Treg percentages was detected in lamina propria (siLP or cLP) and Peyer’s patches respectively. We also examined the percentages of γ-IFN, IL-4 and IL-17 producing CD4 T cells in secondary lymphoid organs and GALT. Because no deliberate immune response was induced in these animals, percentages of cytokine producing CD4 T cells were relatively low (< 5% for IL-4 and IL-17; < 10% for γ-IFN) confirming that CD4 T cell accumulation in GALT was not related to on-going immune responses. We detected increased percentages of γ-IFN and IL-4 producing CD4 T cells in secondary lymphoid organs as previously described but not among GALT CD4 T cells recovered from old animals (data not shown).

Collectively, our data suggest that CD4 T cell increase observed in the GALT reflect progressive recruitment and accumulation of CD4 T cells from lymphoid sites rather than peripheral expansion of gut resident CD4 T cells.

### Age dependent CD4 T cell accumulation in gut associated lymphoid tissue but not in lungs or liver

**Figure 6:**
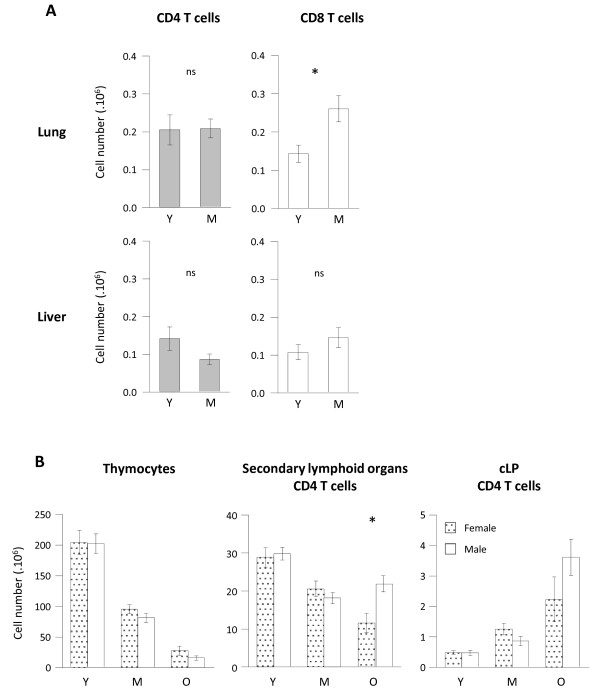
**No T cell lymphopenia in lungs and liver during ageing and no impact of sex on GALT CD4 T cell accumulation. (A)** Lungs and liver CD4 and CD8 T cells were recovered from young (Y) (n = 7), middle-aged (M) (n = 7) C57BL/6 mice. Numeration and FACS staining were performed as previously described. **(B)** Sex dependent analyses of thymocytes (left), pooled secondary lymphoid organs (middle) and colonic lamina propria (right) CD4 T cell numbers. Female animals are represented in dotted columns, male in plain white columns. Cumulative results show the mean ± SEM of absolute numbers recovered from independent experiments comparing young and middle-aged animals. Statistical significance (Student’s *t* test) is shown: ns, non-significant; *, p < 0.05; **, p < 0.01.

We next questioned whether increase in CD4 T cell numbers observed in gut associated lymphoid tissue (GALT) was a common feature of all tertiary lymphoid organs and even of non-lymphoid tissues in middle-aged animals. We thus analysed two additional sites: the mucosa associated lymphoid tissues (MALT) in the lungs and a non-lymphoid tissue: the liver which has been described to comprise important T cell numbers, especially double negative CD4- CD8- T cells [40,41]. To note, T cell recovery in young animals confirmed that GALT represented a major site of CD4 T cell accumulation: CD4 T cell recovery in lungs and liver were approximately 15-fold lower than numbers recovered from small intestine lamina propria (Figure 6A). In the lungs, CD4 T cells were preserved at middle age, whereas CD8 T cell absolute numbers increased, thus demonstrating that lungs MALT differed from GALT. In the liver, no difference among CD4 or CD8 T cell absolute numbers was detected between middle-aged and young mice. Collectively, we demonstrated that CD4 T cell accumulation was essentially restricted to the gut associated lymphoid structures.

### No major influence of sex on CD4 T cell distribution during ageing

Sex of animals and sex hormones may differentially regulate CD4 homeostasis [42]. To ascertain whether CD4 T cell redistribution in cLP occurs equally in male and female animals, we analysed CD4 T cell distribution based on the sex of the animals. As shown in Figure 6B, sex had only minor influence on CD4 T cell lymphopenia at lymphoid sites and CD4 T cell accumulation at mucosal sites. Comparing young, intermediate and old animals, we observed an equal decay of thymocytes in male and female animals. In pooled secondary lymphoid organs, CD4 T cells decay was observed in both groups, although significantly more exacerbated in female animals (p 0.0156 when comparing old groups). Conversely, CD4 T cell numbers recovered in cLP were equally increased in male and female animals. These data demonstrate that CD4 T cell accumulation in gut associated lymphoid tissue associated to CD4 T cell lymphopenia during ageing occurs both in male and female animals, although the amplitude of CD4 T cell depletion in lymphoid organs appeared to be more drastic among female mice.

## Discussion

Ageing is a multifactorial process [24,25] integrating general metabolic changes and immune specific alterations such as progressive decrease in thymic export [21-23]. An expected consequence of declining T cell production is the development of progressive T cell lymphopenia. The incidence of T cell lymphopenia during human ageing is extensively reported in blood samples [11-13] but a more controversial picture emerges from lymphoid organs in murine studies. T cell numbers recovered from spleen or pooled secondary lymphoid organs are reported to be unchanged [24,39] or reduced [14,15] depending notably on the organs considered. In that respect, our current analysis in C57BL/6 mice reconciles this apparent contradiction regarding cell numbers recovered from secondary lymphoid organs: mesenteric lymph nodes and spleen being essentially preserved, whereas superficial lymph nodes studied exhibited significant T cell depletion affecting primarily CD4 T cell in middle-aged animals but also CD8 T cells at older ages. A second common observation regarding immune ageing is the differential behaviour of CD4 and CD8 T cell compartments when ageing developed [43]. Evidence of T cell lymphopenia differently affecting CD4 and CD8 T cell compartments is also described in bone marrow transplantation protocols or in a model of genetic thymectomy [15,34,35]. These differential behaviours of CD4 and CD8 T cells are also substantiated by phenotypic and molecular analyses [28,36,44]. Numerous publications attribute such differential behaviour to specific age related oligoclonal expansion of CD8 T cells both in mice and humans [45-48]. Accordingly, we observed that CD4 T cell numbers recovered from secondary lymphoid organs were rapidly affected: a significant decay was detected in middle-aged animals, whereas CD8 T cells were essentially preserved. However, we demonstrated here that differential behaviour of CD4 and CD8 T cells also arise among their naïve T cell compartments. The size of the naïve T cell compartment theoretically results from the combined effect of thymic production, homeostatic control and peripheral activation leading to recruitment of T cells from the naïve pool. Because loss of thymic production observed during ageing affects equally single positive CD4 or CD8 thymocytes production [21], our results suggest that naïve CD8 T cells are preserved due to poor turn over, whereas naïve CD4 T cell pool appears highly stimulated and directly impacted by partial reduction in thymic production. This faster consumption rate of CD4 T cells may reflect the more pleiotropic function of CD4 T cells compared to CD8 T cells, CD4 T cells being central partners of innate, humoral and cytotoxic responses.

A third classical feature of T cell ageing is a shift from naïve towards effector/memory T cell predominance observed in blood and lymphoid organs [49]. This shift may rely on either naïve T cell decay and/or increase of the effector/memory fraction. By providing naïve and effector/memory absolute numbers, we demonstrated that a different balance between naïve decay and increase in effector/memory cells seems to develop among CD4 and CD8 T cells. In middle-aged animals, naïve CD4 T cell decay was prominent whereas effector/memory CD4 T cell numbers were, albeit significantly, only mildly increased. To note, we excluded regulatory T cells, a particular subset of CD4 T cells exhibiting suppressive activity, from naïve and effector/memory CD4 T cell pool analysis. In contrast, CD8 T cell numbers were essentially preserved in middle-aged animals: no significant difference was detected when considering total, naïve or effector/memory CD8 T cell numbers separately. In old animals, a different picture emerged: a prominent increase in effector/memory CD8 but not CD4 T cells was detected. Our data suggest that changes in activation profile is essentially related to decrease in naïve T cell numbers when considering CD4 T cell compartment, whereas oligoclonal CD8 expansion occurring in older mice also contributes to naïve towards effector/memory shift among CD8 T cell compartment. Collectively, we demonstrated the existence of an unparalleled impact of ageing on naïve CD4 and effector/memory CD8 T cells.

More importantly, we provided in this report the first evaluation of the impact of ageing on CD4 and CD8 T cells residing in secondary and tertiary lymphoid organs and in non-lymphoid tissues. In striking contrast to secondary lymphoid organs, we detected a progressive accumulation of CD4 T cells in all gut associated lymphoid tissues (GALT) considered: Peyer’s patches, lamina propria from the small intestine and the colon. Such increase was not detected for CD8 T cells in Peyer’s patches but was observed at a lesser amplitude in lamina propria. CD4 T cell numbers recovered from lamina propria were consistently up to 6-fold higher than CD8 T cell numbers recovered. Thus, although both CD4 and CD8 T cells appear to accumulate in the lamina propria with age, such effect may be particularly crucial for the CD4 T cell compartment. As a control, we next analysed CD4 and CD8 T cell accumulation in lungs and liver. CD8 but not CD4 T cell accumulation was detected in the lungs mucosa associated lymphoid tissues suggesting CD4 T cell accumulation in the intestine was a specific feature of the GALT. Analyses of T cell recovery in the liver revealed essentially preserved T cell numbers although later time points may be required to fully ascertain the impact of ageing on liver resident T cells. This observation is in accordance with previous publications demonstrating a high increase of DN T cell rather than CD4 or CD8 T cells in aged liver [40,41]. Thus, CD4 and CD8 T cell distribution/accumulation during ageing appears highly differing depending on the tissues considered. The mechanisms responsible for such predominant age dependent accumulation of CD4 T cells in the GALT remain to be further investigated. Two main hypotheses can be formulated: CD4 T cell accumulation in the intestine may reflect age related skewing of CD4 T cell distribution from secondary lymphoid organs to the GALT or specific proliferative activity of CD4 T cells residing in the gut ensuring local CD4 T cell production. Evaluating Ki67 expression in GALT CD4 T cells provided insight showing that local proliferation is not significantly increased but even reduced during ageing. This observation suggests that change in T cell numbers in the gut are predominantly induced by recruitment rather than local proliferation. Accordingly, preserved proportion of FOXP3+ regulatory T cells suggests that CD4 T cell accumulation applies to both conventional and regulatory T cells. Additionally, we detected an association between the severity of T cell lymphopenia at lymphoid sites and CD4 T cell accumulation in colonic lamina propria (cLP). The absence of association with small intestine lamina propria (siLP) may reflect either the lower fold of CD4 T cell accumulation in siLP although different mechanisms of accumulation developing in cLP and siLP respectively cannot be excluded. Increasing numbers of reports demonstrated the crucial role of microbial colonization of mucosal site and mucosal immunity on immune responses [50,51]. It is tempting to speculate that mucosal immunity may directly influence the severity of lymphoid T cell lymphopenia. However, we cannot strictly discriminate in our experimental settings the causal or consequential link between lymphoid T cell lymphopenia and mucosal accumulation. This question will require further investigations.

Collectively, analyses of T cell recovery in multiple sites indicate that T cell lymphopenia is not a consistent feature of ageing: T cell lymphopenia was essentially restricted to the CD4 subset in some secondary lymphoid organs. Such heterogeneity questions the exact incidence of T cell lymphopenia developing during ageing. One may consider that redistribution of CD4 T cells rather than T cell lymphopenia is the crucial phenomena occurring during ageing.

Finally, these observations are also highly relevant in context of accelerated ageing. For instance, HIV infection is frequently defined as accelerated ageing, due to persistent T cell lymphopenia and skewed naïve to effector/memory phenotype. However, HIV infection is associated to drastic CD4 T cell depletion in the gut prior to the progressive depletion detected in the blood [52]. Such observation constitutes a major drawback to the characterization of HIV infection as an accelerated ageing process. One may question the long-term thread that local CD4 T cell depletion in the gut may induce in old HIV infected patients.

## Conclusion

Our results demonstrate that T cell lymphopenia commonly associated to secondary lymphoid organs is surprisingly restricted to these specific sites, and is not applicable to important sites of T cell accumulation such as gut associated lymphoid tissue (GALT). These results are in accordance with a high degree of compartmentalization among organs previously described [53,54]. Ageing appears to induce tissue-specific modulation of T cell compartments: ageing was associated with significant increase in CD8 T cells in lungs, mixed CD4 and CD8 T cell accumulation in GALT, and no change in the liver. Marked decay of CD4 T cells in secondary lymphoid organs during ageing may rely on different sites of CD4 T cell accumulation rather than generalized CD4 T cell decay. Such observation refreshes the concept of age related T cell lymphopenia by favouring a model of age related change in T cell distribution among secondary, tertiary and non-lymphoid sites. The concept of tissular heterogeneity of ageing may also provide an interesting rationale for the diverse effects of ageing on immune responses.

## Methods

### Mice

6 to 8 weeks old C57BL/6 mice were purchased from Janvier Laboratories, and mated in our facilities. Mice were sacrificed at 2 to 6 months of age, 10 to 14 months of age and at 22 to 26 months old respectively named “young”, “middle-aged” and “old” animals. Mice were maintained under pathogen free conditions at the central animal facility of Paris-Sud Faculty of Medicine. As control, young (2 months, n = 6) and middle aged B6-SJL F1 animals (11 months, n = 6) were also analysed. All protocols were conducted in compliance with French and European animal welfare regulations (agreement B-94-043-12 and license 94–440, delivered by the French veterinary authorities) and validated by the Ethics Committee of University Paris-Sud (CEEA27).

### Lymphoid organs cell purification

#### Standard procedures

Single cell suspensions were prepared from lymph nodes (mesenteric and pooled inguinal, brachial, axillary), spleens, thymus and Peyer’s Patches in DMEM High Glucose containing 10% FCS and 20 mM Hepes buffer (PAA Laboratories GmbH) by dilaceration on 70-μm-mesh cell strainer (BD biosciences).

#### Cell isolation from small intestine and colon lamina propria

Intestines were treated following standard procedures [55]. Intestines were washed with PBS 1X, mesentery and fat were removed. Peyer’s patches from small intestine were excised and treated as described above. Intestines were then opened longitudinally, cut into 1 cm pieces, and washed 5 times for 10 min at 37°C in pre-warmed PBS 1X containing 3 mM EDTA. Intestine pieces were then washed for 10 min at 37°C in pre-warmed PBS 1X. The tissues were digested in collagenase 100 U/mL (Sigma) and DNase 50 U/mL (Sigma) in pre-warmed RPMI 1640 containing 10% FBS and 20 mM Hepes buffer at 37°C for 45 min. Aspirating 10–15 times the suspension using a 10 mL syringe completed the digestion. All incubations were done under magnetically agitation. Cell suspensions were washed twice in DMEM High Glucose containing 10% FCS and 20 mM Hepes buffer before flow cytometry staining.

#### Lymphocyte isolation from liver and lungs

Liver single cell suspensions were collected in DMEM High Glucose containing 10% FCS and 20 mM Hepes buffer (PAA Laboratories GmbH), following dilaceration on 100 μm mesh cell strainer. Lungs were washed with PBS 1X, finely minced and stirred in PBS 1X with 400 μg/mL Liberase (Roche) during 20 minutes at 37°C under 250 rpm agitation; suspensions were subsequently filtered and dilacerated on 70 μm mesh cell strainer. Finally, Ficoll centrifugation at 2500 rpm was performed for 20 minutes to isolate lymphocytes. These lymphocyte suspensions were washed twice in DMEM High Glucose containing 10% FCS and 20 mM Hepes buffer before flow cytometry staining.

### Absolute numbers determination

Cells counts were performed in duplicates after addition of Trypan blue dye using Malassez haemocytometer cell.

### Flow cytometry

Extracellular staining was preceded by incubation with purified anti-CD16/32 antibodies (FcγRII/III block, 2.4G2) (eBioscience) to block non-specific staining. Cells were stained with FITC-, PE-, PerCP-Cy5.5-, PE-Cy5-, PE-Cy7-, APC-, and APC-H7- labelled appropriate antibodies including: TCRβ (H57-597, eBioscience); CD45 (30-F11, eBioscience and BD Biosciences); CD44 (IM7, eBioscience); CD8α (53–6.7, eBioscience); CD62L (MEL-14, eBioscience); CD4 (GK1.5, BD Biosciences and Miltenyi Biotec); or appropriate isotype Abs. Intranuclear FOXP3 staining was performed using eBioscience PE- or APC-conjugated FOXP3 staining buffer set (FJK-16 s). Intranuclear Ki-67 staining was performed using BD Pharmingen Ki-67 (B56) on permeabilized cells. Six-colour flow cytometry was performed with a FACSCanto cytometer (BD Biosciences). Data files were analysed using FlowJo software (Tree star Inc.).

### Statistical analyses

Statistical analyses were performed using unpaired Student’s *t* test with Graph Pad Software. Mean and standard error mean of experiments are shown. Association were tested using a Spearman test.

## Competing interests

The authors declare that they have no competing interests.

## Authors’ contributions

KM performed the experiments, analysed data and contributed to experiments designing and manuscript writing. SB ensured mice welfare during the course of the work. CB designed the experiments, analysed the data and wrote the article. All authors read and approved the final manuscript.
